# Nerve Counterfeits of Uterine Disease1Read at the meeting of the Buffalo Obstetrical Society, November 24, 1891.

**Published:** 1892-03

**Authors:** Henry D. Ingraham

**Affiliations:** Buffalo, N. Y., Professor of Gynecology, Medical Department, Niagara University; Gynecologist to the Buffalo Hospital, Sisters of Charity; 405 Franklin Street


### Buffalo Medical and Surgical Journal

Vol. XXXI. MARCH, 1892. No. 8.

### Original Communications.

### NERVE COUNTERFEITS OF UTERINE DISEASE.[1]

By HENRY D. INGRAHAM, M. D.,

Buffalo, N. Y.,

Professor of Gynecology, Medical Department, Niagara University; Gynecologist to the Buffalo Hospital, Sisters of Charity.

The object of this paper is to enter a protest, feeble though it be, against the unnecessary or heroic treatment of the uterine organs of women who are suffering chiefly from nervous exhaustion, or a hyperesthetic condition of the nervous system; also, incidentally, to protest against the unnecessary vaginal examination of young unmarried women. I know of no better way to enter my protest, and make myself understood upon these subjects, than to relate, briefly, a few of the many cases which I have seen; and were it not for the cases which have occurred in my own practice, I would not believe that intelligent physicians, some of them giving especial attention to the diseases of women, would recommend, in many instances, the treatment they do. Doubtless, many general practitioners are more inclined to resort unnecessarily to local treatment than the specialist. As far as I know, Prof. Goodell is the only writer of a text-book upon the diseases of women who devotes any particular attention to the Nerve Counterfeits of Uterine Diseases, as he designates the class of cases to which reference is made. Yet many of the best men in the country, through the columns of the medical journals, have protested against the local treatment of the female generative organs, and the mutilation of women when there is no organic lesion. One would think that enough had been said upon these subjects so that cases like the ones about to be related would be unknown, yet they occur frequently even at the present day.

Case I. About three years ago I was called, in consultation, to see Mrs. A., aged thirty-eight, married eighteen years, one child seventeen

1. Read at the meeting of the Buffalo Obstetrical Society, November 24, 1891.

years old. Menstruated first at fourteen, regular, but always suffered from dysmenorrhea. Never had rugged health, but has been worse since the birth of her child; had pains in both iliac regions, more marked in the right; pains in the back, extending down the thighs, in back of neck and head, with dyspeptic symptoms and constipation. The dysmenorrhea had been worse since the birth of the child, and much worse for the past four or five years. She had severe pains in the back and sides one week before the menstrual period, during the period, and a week after, so that she was comfortable only about one week in each month. Upon vaginal examination, the uterus was found to be slightly retroflexed, yet not enough to account for the dysmenorrhea or other pains. There was no disease of the appendages, as far as could be discovered.

The patient had been under the care of several physicians, and had received all manner of treatment. She was formerly, for a long time, a patient of the late Prof. White. Her present physician had attended her for a year or more, and he fully believed if her ovaries were removed she would regain her health. After a thorough examination, I concurred in his opinion. A few weeks later she entered the Buffalo Hospital of the Sisters of Charity, and I removed her tubes and ovaries. Neither were diseased to any extent. She made a good recovery, ceased to menstruate, and for several months was quite well, but for the past two years her health has been but little better than before the operation, except she does not have the dysmenorrhea, because she does not menstruate; otherwise her condition is very much as before the operation.

Case II. Mrs. L. E., aged thirty-nine, American, married seventeen years, no children. Menstruated first at fourteen, usually regular, occasionally slight dysmenorrhea. Health good until five years ago, when it began to fail. She had pains in the back, both iliac regions, more especially the left, the hypogastrium, and head. Menstruation somewhat painful, had leucorrhea, bowels constipated, appetite poor, and restless at night. Was able to do the greater part of her housework, her family consisting of herself and husband. She had been treated by several physicians for various diseases, for three years, without any marked improvement, sometimes better, at other times worse. Two years ago the first of last January, she had her tubes and ovaries removed by a celebrated gynecologist, and I understand there was no morbid change to be discovered in these organs by the naked eye, after their removal. She made a good recovery from the operation, and for a few months was much improved, yet not free from pain in the back and left iliac region at any time. Six months after the operation she was worse than before it was performed, was confined to her bed, able to sit up only a few minutes at a time. The surgeon who operated now concluded that she had some spinal disease, and called to his aid an orthopedic surgeon, who put her into a sole-leather jacket. Four months later she was no better, and I was called to see her. I found her in bed, unable to sit up on account of the pain in her back and left iliac region. A thorough examination revealed no soreness, tenderness, or deformity of the spine whatever, and it was said that there never had been any. She complained of severe backache, yet she did not flinch under very firm pressure. Pressure in either iliac region caused her to flinch very quickly, unless her attention was called to something else, when she could endure quite firm pressure without noticing it. The uterus was movable, and although she said she had profuse leucorrhea there was no evidence of it, nor any condition of the parts that would indicate its existence. In fact, no lesion could be discovered anywhere. She was informed, if she would go to a hospital and allow a certain treatment to be faithfully carried out, that she would get well. It was intended to give her the Mitchell treatment for nerve exhaustion, but when she was informed what that would be she declined to go, saying that she could not endure it, and that her husband had spent all his money, and she could not afford to go. She wished me to attend her at her house, but I declined, believing that I could do her no good if she remained at home. It is now fourteen months since I saw her, she having left the city. The following is a description of her present condition, furnished by a sister-in-law, with whom she is living, the patient not being able to write. She says:

"While it was warm enough so that Mrs. E. could sit out doors she sat up quite a little, but since it is cooler she does not have strength to sit up scarcely any. The pain in her back and left side is no better than when you saw her. She has a pain and very hot feeling in the top of her head, running down into her face and back of her neck, nearly all the time. Her appetite is good, but her food distresses her, so she has to be very careful what she eats. She still wears the jacket and can't leave it off long enough to take a bath without being worse for two or three days. Has cold feet and legs; can't keep them warm. Black specks float before her eyes all the time. Bowels constipated."

CASE III. Miss C. N., aged twenty-two, born in Canada. This patient is a beautiful, highly educated, and accomplished young woman, of medium size and well developed. Always had good health until a little more than a year ago. Menstruated first at fourteen; was somewhat irregular, and the flow was scanty a part of the time, but never suffered from dysmenorrhea. Has been in school continuously. In her twentieth year she was sent to a "young ladies' college," her room being on the sixth floor. She took five and six studies, including music. After a few months her health began to fail. She had headache, pain in the back, also both iliac and hypogastric regions; appetite poor, bowels constipated ; slept poorly ; menses more irregular and scanty than formerly, and finally she did not menstruate for four months.

At last vacation came, and she was sent into the country to recuperate. She improved somewhat, but was unable to enter school at the beginning of the next term. Later she was considerably better, and, being anxious to finish the course, she again entered school, but had not been there long before her health failed entirely, and she left completely broken down. It is the same old story,—too much study and too little attention to the plainest and simplest hygienic laws. She went home and was under the care of the family physician for some time, but did not improve. As she was suffering from pain in the head, neck, and back, and also from severe paroxysmal pains in either iliac region, impaired appetite, and an extremely nervous condition, complicated with amenorrhea, a physician was called in consultation who does some uterine surgery. After examining the patient, he informed the friends that she had diseased tubes and ovaries, and that the only relief for her was the removal of the offending members. To this the family physician agreed, but the mother objected. The suffering of the patient, from pain in either iliac regions, was terrible. She did not improve, and as the consultant continued to say to the mother, "I told you so," she finally gave her consent for the removal of these organs, and the day for the operation was set. As the time approached, although the patient was no better, the mother changed her mind, and refused to have the operation performed. There was no improvement in the case, and a little later another gynecologist was called. After a thorough examination, he declared that there was no trouble whatever with the tubes and ovaries, but that the poor sufferer had retroflexion of the uterus, complicated with stenosis of the internal os, and endometritis. With the consent of the attending physician and the mother, he forcibly dilated the cervix, under an anesthetic, treated the endometritis, replaced the uterus, and introduced a pessary to retain it in position. Still the patient did not improve; the pains continued as severe as ever. Every afternoon, between three and seven o'clock, they began, sometimes earlier, sometimes later, and lasted well into the night, the patient sleeping but litte until three or four o'clock in the morning. When I saw her, the latter part of last August, she had been in bed over three months, was unable to walk, and only able to sit up a few minutes at a time ; had no appetite, bowels constipated, yet her tongue was clean, and she had no fever. She still had the same terrible pains in either iliac region, radiating to her back and down her thighs. After a most thorough and careful examination of all the organs, I was unable to discover any trouble whatever with the uterus, tubes, or ovaries, or, in fact, any organic lesion anywhere. The uterus was slightly smaller than normal, but there was no retroflexion, although the pessary, which had been introduced some time before, could not be borne, and was removed by the family physician three or four days after its introduction. It was noticed that, although the patient had severe pain in either iliac region, and that pressure could not be tolerated, yet, when her attention was diverted, stronger pressure could be made. As she had taken nearly all the anodynes, nervines, and many of the tonics of the pharmacopeia, it was thought best to medicate her as little as possible. Bromides, with chloral, were advised for the pains and sleeplessness ; iron, combined with vegetable tonics, to improve the general condition ; also the use of electricity, not so much for any specific effect that it might have, but as a nerve tonic. The patient was assured that the use of electricity would, in a short time, control the pains, and that the result would be accomplished much sooner if she made a strong effort to control the pains as far as possible. Systematic feeding was advised, especially to give at regular intervals a certain quantity of milk, so that during the day she should take at least a quart, this amount to be increased, so that in a week or ten days she should take double the quantity. Later on, eggs, beef tea, and other light nourishing diet, to be given in generous quantities. Her bowels to be kept in proper condition. Under no circumstances was she to sit up, except to have her bed made, until the disappearance of the pains. Visitors were not allowed. Her mother, who acted as nurse, was advised to notice her pains as little as possible, and to cheer, by word and example, all she possibly could. This treatment, with slight variations, was faithfully carried out, not because the attending physician had much faith in it, but because every other treatment advised, except the removal of the tubes and ovaries, had been tried and failed, and this was a last resort. In three weeks the patient was free from pain and out of bed. Since then she has steadily improved. At this writing, three months from the time I first saw her, she says she never felt better. She has menstruated twice, freely, without pain, has been out on the street for several weeks, and undertakes any reasonable exertion. She has gained twenty pounds, and hardly looks like the same person.

Several other cases might be reported that I have seen, similar to the ones given, but these are enough to demonstrate the points claimed.

Patient number one had been under the care of several very excellent physicians for a number of years, yet she did not improve, and it was thought if the menopause was brought about her health would improve. It would certainly be to her advantage to be relieved of the dysmenorrhea from which she suffered so severely. For a few months after the operation she was much better, but family troubles occurred, and from that time she was worse, the old pains in the back and iliac regions returned, and now she is almost as much of an invalid as before the operation, yet she does not suffer as severely, as she has no dysmenorrhea, but the benefit derived from the operation was much less than was expected, both by her medical attendant and myself.

Patient number two had always been healthy until three years before her operation, when she began to fail. She had been married a number of years and had no children; she had pains in her back and sides, so she thought there must be some disease of the reproductive organs. She consulted a physician who believed he discovered diseased tubes and ovaries, and treated her accordingly, but she did not improve, and she consulted others, each finding something different, and telling her that he could relieve her. By far the most competent physician whom she consulted before her operation, told her, after making a thorough examination, that she had no organic disease of the reproductive organs whatever, and that local treatment was doing her more harm than good, that she needed altogether different treatment, and that she could get well. This evidently did not please the patient, as she did not consult him again. After the removal of the tubes and ovaries she improved, because she believed she was relieved of the cause of her trouble, and was elated, thinking from that time on she was to be a well woman. After a time reaction occurred, and she was worse than ever. Then some form of spinal disease was discovered and the sole leather jacket applied. It may be possible for a patient to have some serious disease of the spine without any tenderness or deformity, yet the writer does not believe it. But if it is, the continuous use of a sole leather jacket for eighteen months should at least benefit the patient somewhat if there is any virtue in the jacket. Before the operation the patient did her housework and attended to all her duties, although a partial invalid. Today she is bed-ridden, suffers vastly more pain than before the operation, is able to sit up but very little, cannot walk, and never expects to be any better. Does any one say this patient was improved by the operation?

Patient number three simply broke down from overwork. Her parents and teachers did not realize the fact which should be understood by all who have charge of the young, that the physical development is impaired when the mental faculties are overworked; that the growing girl cannot overstudy, keep late hours, or go beyond her strength in any way, especially at the menstrual period, without disastrous results. This young woman's nervous system was unduly strained, then came the explosion. It came early enough so that no serious results are likely to remain permanently, except there is no doubt that she will be more nervous in after life than she would have been if this trouble had never occurred. Yet the removal of this young woman's ovaries would have been an irreparable loss, entirely unjustifiable, and possibly one that would have grieved her beyond endurance in later years. Why any intelligent physician should overlook the real disease and accuse a normal ovary as the cause of the trouble is beyond the writer's comprehension, unless he wished to add to the number of his operations without any regard to the well-being of the patient. Neither do I understand why it was necessary to dilate the cervix under an anesthetic, make a half dozen or so applications to the cavity of the uterus, and introduce a retroflexion pessary, which was removed within four days because it could not be tolerated. It does not seem that this treatment was necessary when the patient had never suffered from dysmenorrhea, leucorrhea, or any other symptoms, except pain, that would indicate disease of the uterus or its appendages. Surely such treatment would not restore a retroflexion of the uterus, nor cure an endometritis, that would not disappear under general treatment. I would even go further and say that we are not justified in making a vaginal examination in young unmarried women, unless all other means for their relief have been tried, or there is some urgent necessity for it. Prof. Goodell says "it is committing a moral rape," a sentiment that must be endorsed by all noble-minded physicians. At the present time I have three patients under my care who have been told by competent physicians that their tubes and ovaries must be removed before they could ever be any better. One patient has almost entirely recovered her former health, and the others are well on the road towards good health. I have had several similar cases, besides many who have been treated for displacements, chronic hyperplasia or metritis, endometritis with leucorrhea and dysmenorrhea, who recovered with but little or no local treatment. The symptoms of nerve exhaustion are much the same as those of uterine disease, especially if the woman has any knowledge of the symptoms of real derangements of the uterus. Yet, when the symptoms are out of all proportion to the local lesions, it is usually a pretty sure indication that the trouble is general, not local, and that the treatment to be effective must be general. Then, again, the history of the case will do much to clear up the diagnosis if it be at all doubtful.

Many are the causes of nerve exhaustion and its ally, hysteria, with its counterfeited symptoms, the most common of which are anything that produces worry or grief, as excessive and prolonged mental strain, whether it be in the school girl, the school teacher, the mature maiden, or the wife, disappointments in love, ill-mated and unhappy marriages, reverses of fortune, or the failures and disappointments of life. "Life is big with shattered hopes and wrecked ambitions, and life is, therefore, full of wrecked and shattered nerves." The climacteric with its nerve disturbances, sterility and sexual excess, are each responsible for causing the disease. And when the general health is impaired, neurasthenia or hysteria are liable to fasten themselves upon sensitive or slightly diseased portions of the body, especially upon the reproductive organs, which hold an emotional kinship with the brain.

From the hyperemia and dysmenorrhea developed by the neuroses we may get secondary structural changes, such as hyperplasia, endometritis, and displacements, which need local treatment in addition to general measures, yet in many cases local treatment is not only unnecessary but positively injurious, and should not be resorted to. With young unmarried women we should not even make an examination of the uterine organs until after a thorough and exhaustive trial of general treatment for these conditions. The proper treatment for cases like the ones mentioned is that recommended by Prof. S. Weir Mitchell, with such variations as are indicated in each individual case.

Prof. Goodell says: "The crying medical error of the day is, in my opinion, the mistaking of nerve disease for womb disease."

405 Franklin Street.

---

No end of people suffer from bad food and do not know it. They do not get nourished. The food is often more than sufficient in quantity, but is very deficient in quality, and appears to be cooked by persons absolutely without sense. Cooking schools ought to be multiplied, and those who suffer in pretentious places from cold food which ought to be hot, and tasteless beef which ought to be good, should speak out, so that the steward and managers will remember that their customers are disgruntled, and never mean to come into the place again until things are changed.—*Post Graduate.*

